# Durable complete remission with aromatase inhibitor therapy in a patient with metastatic uterine carcinosarcoma with poor performance status and coagulation disorders: a case report

**DOI:** 10.1186/s13256-017-1262-y

**Published:** 2017-04-19

**Authors:** P. Martin-Romano, M. Jurado, M. A. Idoate, L. Arbea, J. L. Hernandez-Lizoain, D. Cano, J. A. Paramo, S. Martin-Algarra

**Affiliations:** 1 Department of Oncology, Clínica Universidad de Navarra, Avenida Pío XII 36, 31008 Pamplona, Spain; 2 Department of Gynecology, Clínica Universidad de Navarra, Pamplona, Spain; 3 Department of Pathology, Clínica Universidad de Navarra, Pamplona, Spain; 4 Department of General Surgery, Clínica Universidad de Navarra, Pamplona, Spain; 5 Department of Radiology, Clínica Universidad de Navarra, Pamplona, Spain; 6 Hematology Service, Clínica Universidad de Navarra, Pamplona, Spain

**Keywords:** Uterine carcinosarcoma, Hormonal receptors, Aromatase inhibitors, Complete pathological response, Long-term survivor

## Abstract

**Background:**

Chemotherapy is considered the most appropriate treatment for metastatic uterine sarcoma, despite its limited efficacy. No other treatment has been conclusively proved to be a real alternative, but some reports suggest that anti-hormonal therapy could be active in a small subset of patients. We report the case of a patient with metastatic uterine carcinosarcoma with positive hormonal receptors and a complete pathological response.

**Case presentation:**

A 54-year-old white woman presented to our emergency room with hypovolemic shock and serious vaginal bleeding. After stabilization, she was diagnosed as having a locally advanced uterine carcinosarcoma with lymph nodes and bone metastatic disease. In order to control the bleeding, palliative radiotherapy was administered. Based on the fact that positive hormone receptors were found in the biopsy, non-steroidal aromatase inhibitor therapy with letrozole was started. In the following weeks, her general status improved and restaging imaging tests demonstrated a partial response of the primary tumor. Ten months after initiating aromatase inhibitor therapy, she underwent a radical hysterectomy and the pathological report showed a complete response. After completing 5 years of treatment, aromatase inhibitor therapy was stopped. She currently continues free of disease, without further therapy, and maintains a normal and active life.

**Conclusions:**

This case shows that patients with uterine carcinosarcoma and positive hormone receptors may benefit from aromatase inhibitor therapy. A multidisciplinary strategy that includes local therapies such as radiation and/or surgery should be considered the mainstay of treatment. Systemic therapies such as hormone inhibitors should be taken into consideration and deserve further clinical research in the era of precision medicine.

## Background

Uterine sarcomas are rare diseases with poor outcomes and a diverse histopathological classification. Taken together, these facts hamper a consensus on risk factors and treatment guidelines [1–3]. Despite its limited efficacy, chemotherapy appears to be the optimal regimen for metastatic disease. However, this approach remains controversial, especially in patients with poor performance status (PS) at the time of diagnosis. On the other hand, targets of the estrogen and progesterone receptor have been successfully employed in small series of patients [4, 5].

Uterine sarcomas account for nearly 3% of all uterine cancers. Histological categorization according to the World Health Organization Classification of Tumours is represented in Table 1 [6]. The most frequent histological type is leiomyosarcoma, responsible for almost 40% of all cases, followed by endometrial stromal tumors, and undifferentiated sarcomas (15% and 5%, respectively) [1, 7, 8]. Malignant mixed Müllerian tumor or carcinosarcoma of the uterus represents almost 40% of uterine sarcomas. Furthermore, it is now contemplated as a dedifferentiated form of endometrial carcinoma with epithelial and sarcomatous/mesenchymal components [1].

**Table 1:** World Health Organization classification of tumors 2003. Pathology and genetics of tumors of the breast and female genital organs. Uterine sarcomas [6]

Mesenchymal tumors	
Endometrial stromal and related tumors	
Endometrial stromal sarcoma, low grade	10–15%
Undifferentiated endometrial sarcoma	5–10%
Smooth muscle tumors	
Leiomyosarcoma	40%
Mixed epithelial and mesenchymal tumors	
Carcinosarcoma (malignant Müllerian mixed tumor; metaplastic carcinoma)	40%

Epidemiology and prognosis are both very different according to each uterine sarcoma subgroup. Leiomyosarcoma and endometrial stromal tumor normally occur in premenopausal women and localized disease is the most common stage at diagnosis. Regardless of the stage at diagnosis, leiomyosarcoma is an aggressive illness with a poor outcome [7, 9] compared to endometrial stromal tumors [10]. Although early stages of both tumors are usually treated with hysterectomy and bilateral salpingo-oophorectomy without lymphadenectomy, there are no standard recommendations for adjuvant therapy or advanced disease treatment due to the absence of randomized clinical trials [5].

Uterine carcinosarcoma (UCS) is typically diagnosed in post-menopausal women and usually presents extrauterine spread at the time of diagnosis. The following were suggested as prognostic factors favoring survival: age <40, white race, early stage disease, lymphadenectomy, and adjuvant radiotherapy (RT) [11]. Patients with UCS present a 5-year actuarial survival rate of approximately 30%, mainly due to the high rates of both local and distant relapses [1]. An association between elevated cancer antigen-125 (Ca-125) levels, extrauterine disease, and poor survival has been suggested in a single institution study [12]. When diagnosed at an early stage, the most accepted approach includes total hysterectomy, bilateral oophorectomy, pelvic and aortic lymphadenectomy, omentectomy, and peritoneal cytology, followed by adjuvant pelvic RT [13]. Lymph node dissection has demonstrated an upstaging in 20% of patients with early stage disease [1]. On the other hand, surgical cytoreduction has been investigated in advanced stage, showing increased survival when patients with UCS underwent a complete resection [14]. The role of adjuvant chemotherapy has been studied in small series of patients. The combination of cisplatin and ifosfamide has shown an improvement in both disease-free and overall survival in resected UCS [8, 15]. For advanced disease, paclitaxel-based regimens have promising results in terms of response rates and long-term survival [16, 17]. A phase III randomized trial of ifosfamide and paclitaxel versus carboplatin and paclitaxel could clarify this issue. On the other hand, adjuvant RT decreases the risk of pelvic recurrence [18]. Although multimodal treatment seems to be the appropriate approach, the optimal combination and sequence has yet to be defined [19].

We present the case of a patient with an unresectable and metastatic UCS with positive hormone receptors, and a poor PS due to vaginal bleeding and a pulmonary thromboembolism, that subsequently improves radically and achieves a complete response with aromatase inhibitor therapy (AIT). Currently, the patient remains free of disease after completing 5 years of treatment.

## Case presentation

The case of a 54-year-old white postmenopausal woman with an unremarkable past medical history except for a tobacco smoking habit and a few weeks of weight loss, weakness, anorexia, and abdominal mass is described.

**Fig. 1:**
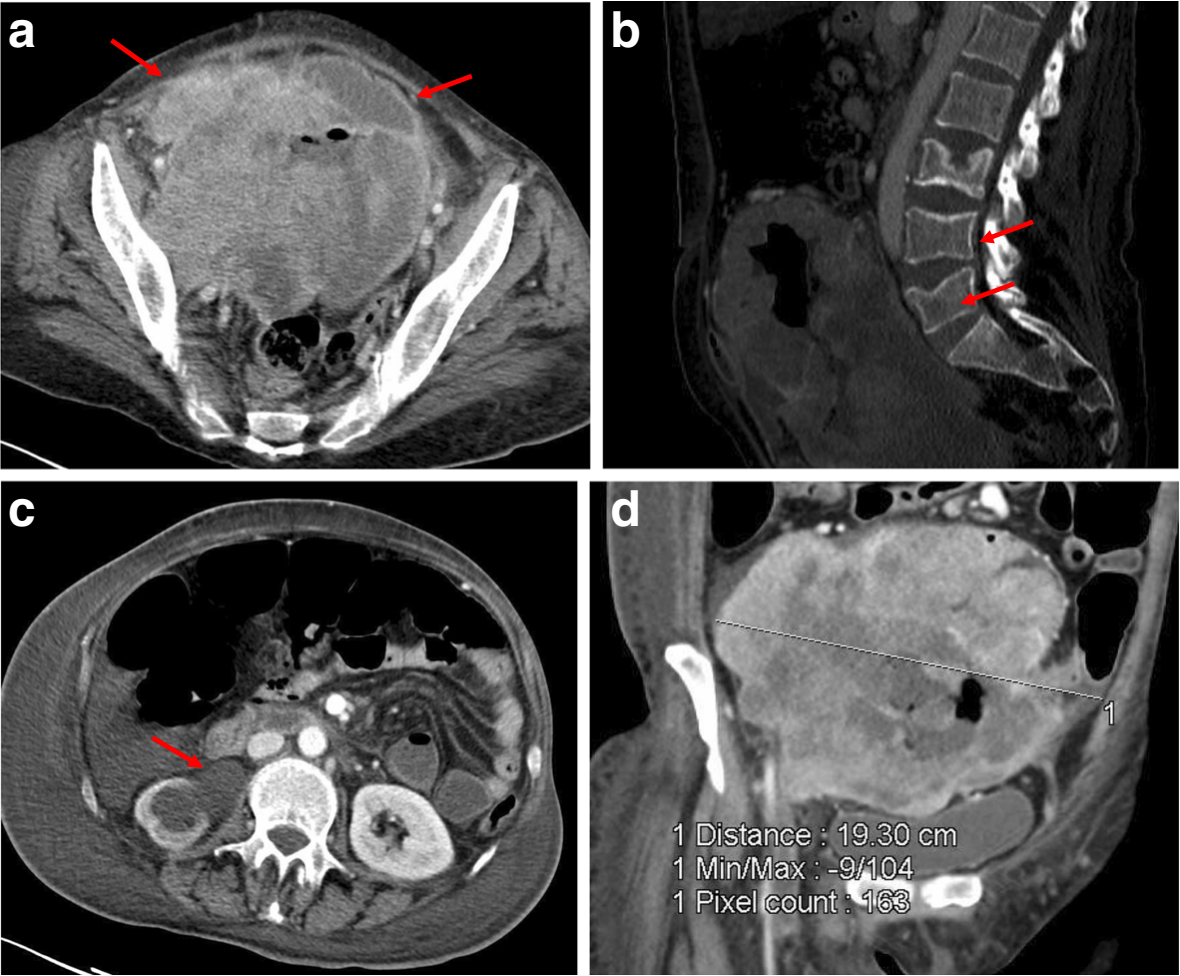
**a** Computed tomography scan at diagnosis showing the pelvic mass (*arrows*). **b** Computed tomography scan at diagnosis showing the bone metastasis in L3 (*arrows*). **c** Computed tomography scan at diagnosis displaying a right hydronephrosis (*arrows*). **d** Computed tomography scan at diagnosis showing the pelvic mass

**Fig. 2:**
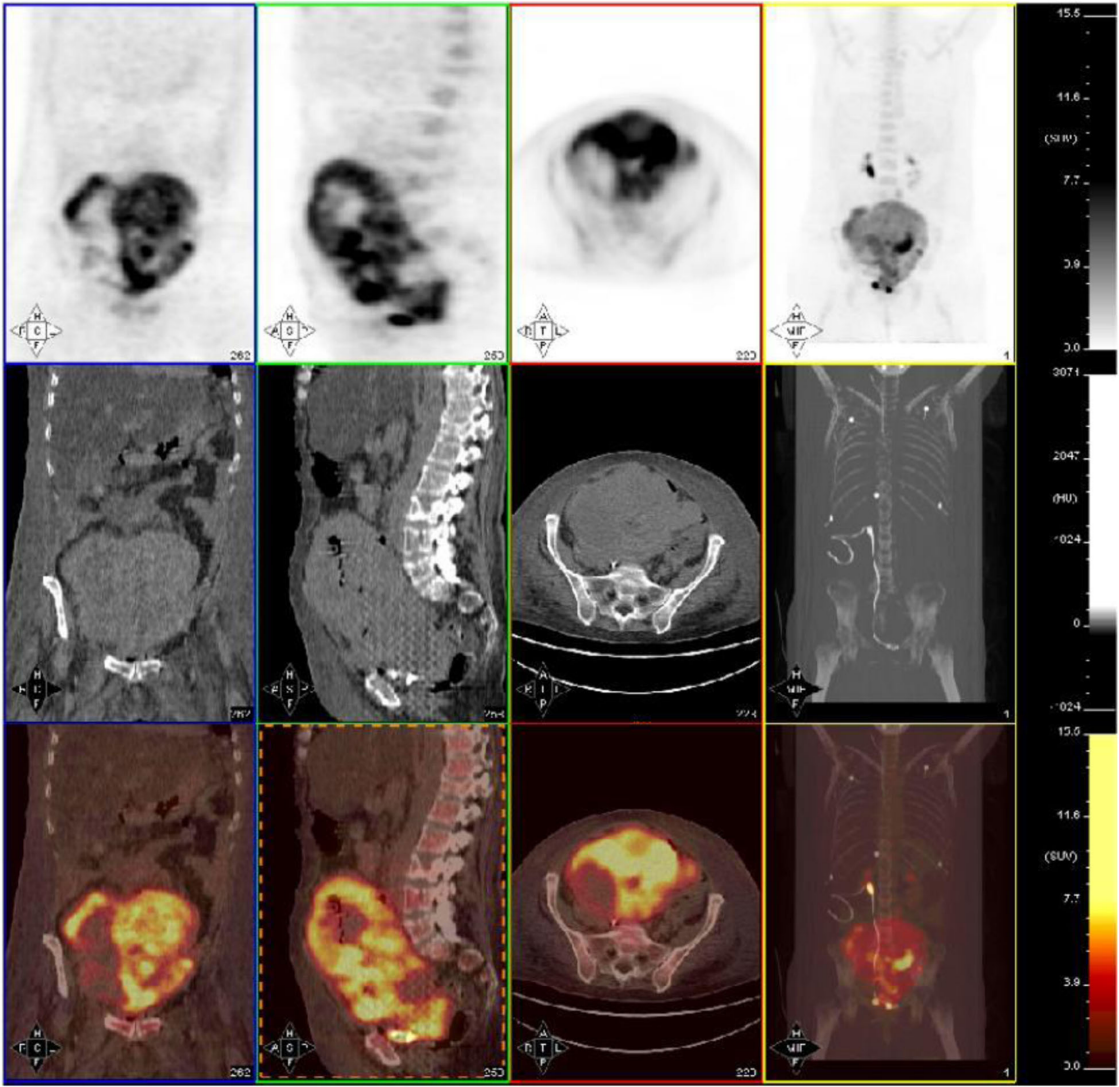
Positron emission tomography scan at diagnosis

**Fig. 3:**
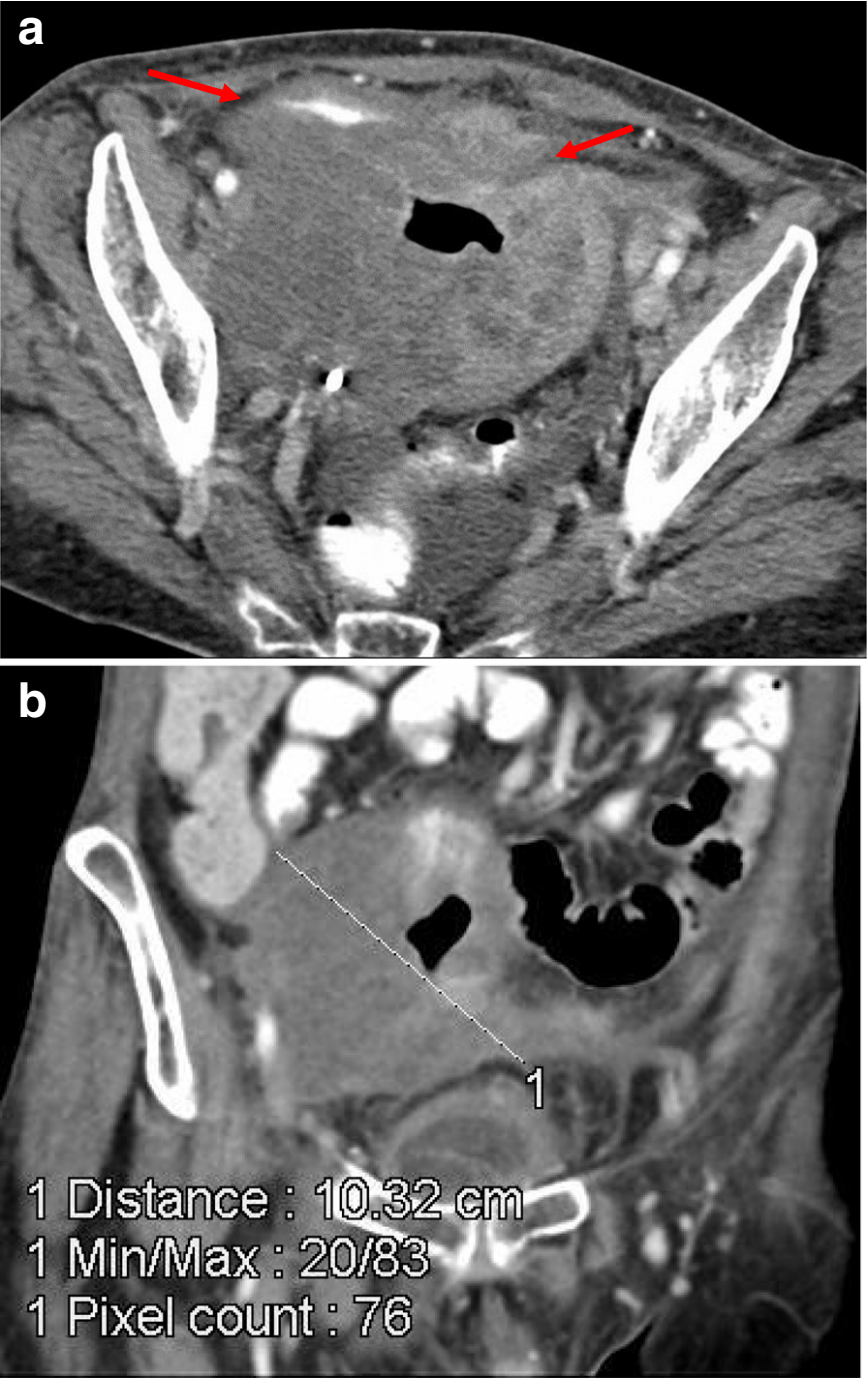
**a** and **b** Computed tomography scan showing partial response after 3 months of treatment. *Arrows* on panel **a** are pointing to the pelvic mass

**Fig. 4:**
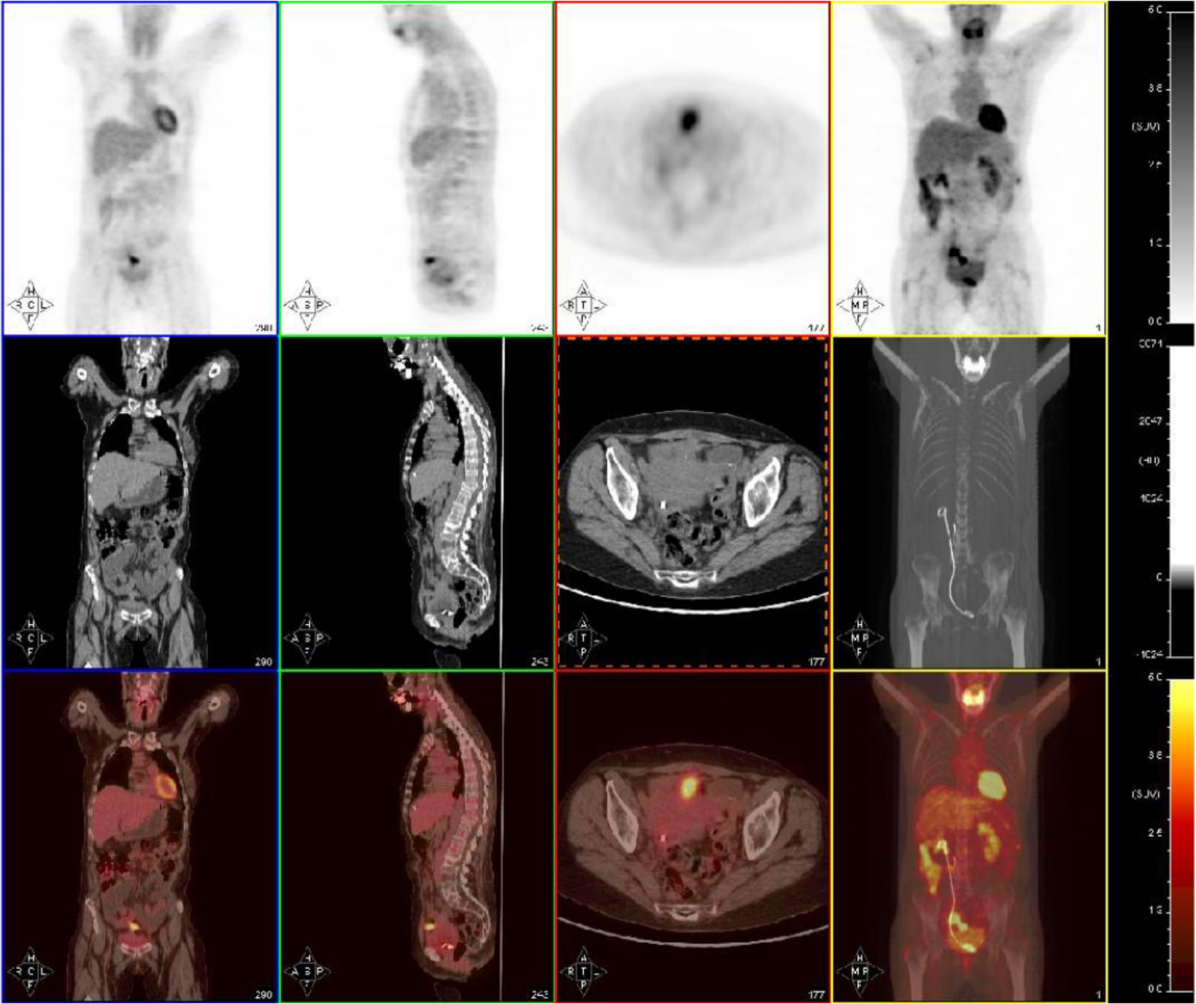
Positron emission tomography scan after 10 months of therapy

The patient presented to our emergency room in April 2009 after a 5-day history of severe vaginal bleeding and progressive dyspnea, with hypovolemic shock, and confusional status. A physical examination showed hypoxemia, a large abdominal mass, intense abdominal discomfort, and left lower extremity edema. A hemogram showed a severe anemia (hemoglobin 5.5 g/dl). An abdominal computed tomography (CT) scan demonstrated a 20-cm ill-defined heterogeneous hypogastria mass that originated in her uterus with several inner bleeding and necrotic areas, invasion of her right ureter, and both her right iliac artery and vein (Fig. 1a, d); para-aortic and left iliac lymphadenopathy along with bone distortion of L3 and L4 vertebrae were also present (Fig. 1b), as well as a right hydronephrosis (Fig. 1c) due to tumor invasion of her distal ureter, and pulmonary thromboembolism. Admission to the intensive care unit was required for continuous monitoring along with fluid resuscitation, blood transfusion, and anticoagulation treatment. A right ureteral stent placement was also required. As soon as she was stabilized, a biopsy of the pelvic mass was carried out. The pathological diagnosis of a high grade endometrial carcinosarcoma was obtained (Fig. 2a). Intense immunoreactivity to vimentin and keratin was observed in 100% of the tumor within the original biopsy (Fig. 2b, c). Immunohistochemical positive results were also detected for estrogen and progesterone (Fig. 2d). A positron emission tomography (PET) scan confirmed the presence of a large, heterogeneous, and highly metabolic pelvic mass with a maximum standardized uptake value (SUV max) of 17.33, with extension to her lower abdominal cavity. Other pathological deposits were present at her left para-aortic ganglia and L3 to L4 vertebral bodies (Fig. 3). At diagnosis, her Ca-125 was 522.8 UI/ml (Fig. 4a), carcinoembryonic antigen (CEA) was normal, and alkaline phosphatase was 767 UI/L.

Given the existence of metastatic disease and an Eastern Cooperative Oncology Group (ECOG) PS of 3, the illness was deemed terminal and non-curable. The patient and her closest relatives confronted the dismal prognosis and, therefore, initiation of chemotherapy was declined.

Due to the presence of a large mass responsible for her vaginal bleeding and severe anemia, as well as the high risk of renal complications as a result of the extrinsic compression of her ureter, palliative radiation was considered. She was then referred to our radiation oncology department for external beam radiation therapy of the abdominopelvic mass, with hemostatic intention. Tridimensional external beam RT with parallel opposed anterior to posterior-posterior to anterior (AP-PA) fields was administered (ten fractions of 2.5 Gy) with complete control of her hemorrhagic symptoms and PS improvement to ECOG 2.

Once the radiation treatment was completed, based on her immunohistochemical findings and prior reports [4, 20–22], treatment with an oral non-steroidal aromatase inhibitor (letrozole 2.5 mg/daily) was initiated. In May 2009, 5 days after the initiation of AIT, she was discharged and scheduled to be followed on an ambulatory basis. On the first visit, after 1 month of AIT, she displayed PS progress to 1 without any side effects, including hot flashes or joint pain.

**Fig. 5:**
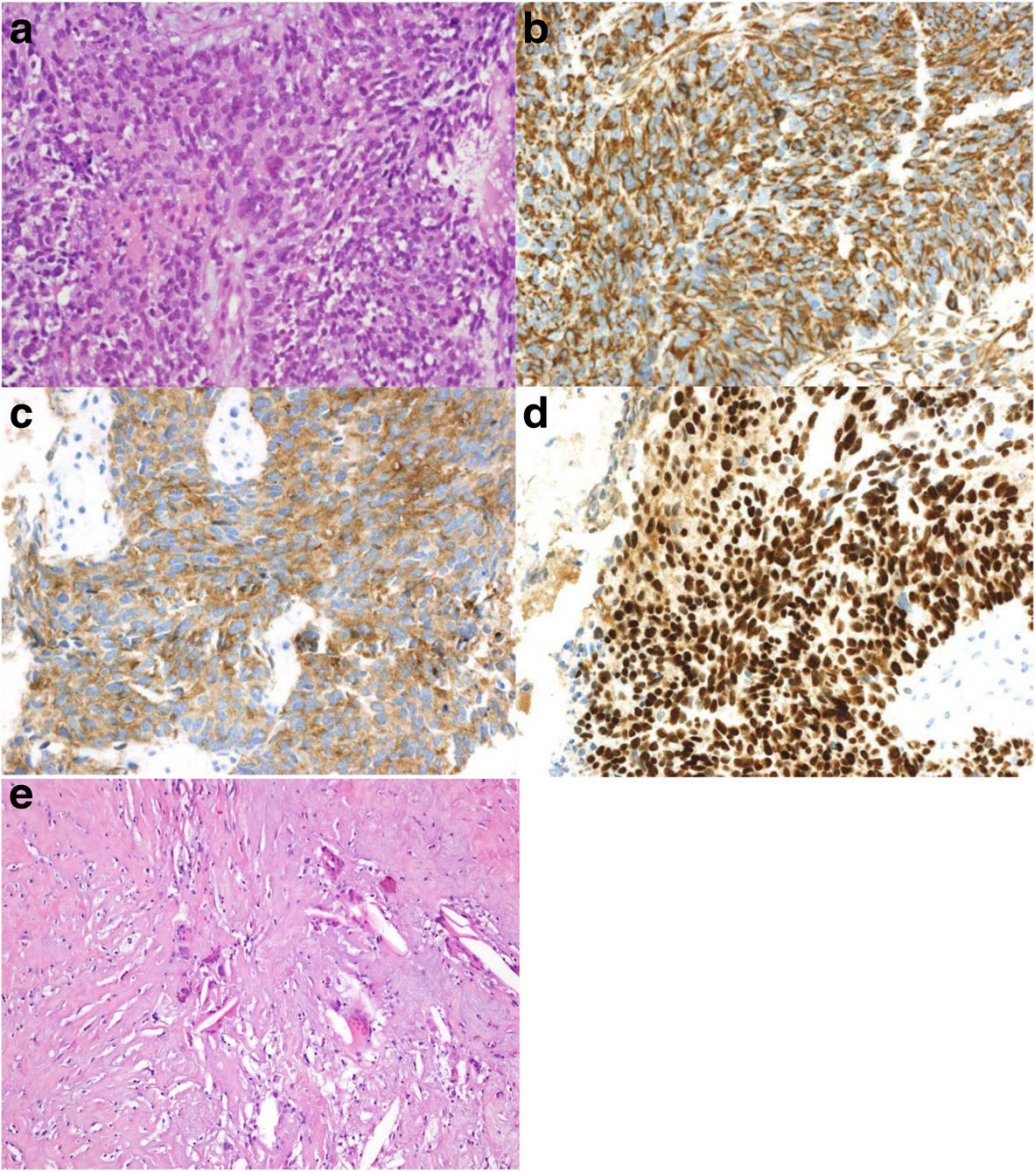
**a** Pathological staging at diagnosis and before treatment. **b** Vimentin expression at diagnosis. **c** Keratin expression at diagnosis. **d** Estrogen expression at diagnosis. **e** Pathologic complete response (ypT0)

Three months after AIT initiation, a hospital admission was required due to urinary infection. An abdominopelvic CT scan demonstrated a partial response (Fig. 5a, b) according to Response Evaluation Criteria in Solid Tumors (RECIST) criteria (7.6 cm, previous 20 cm) and a fistula between her small intestine and vagina. Given the good radiological response, and her general improvement, an intestinal bypass was performed and AIT was maintained at the same dose for 8 additional months, without side effects.

**Fig. 6:**
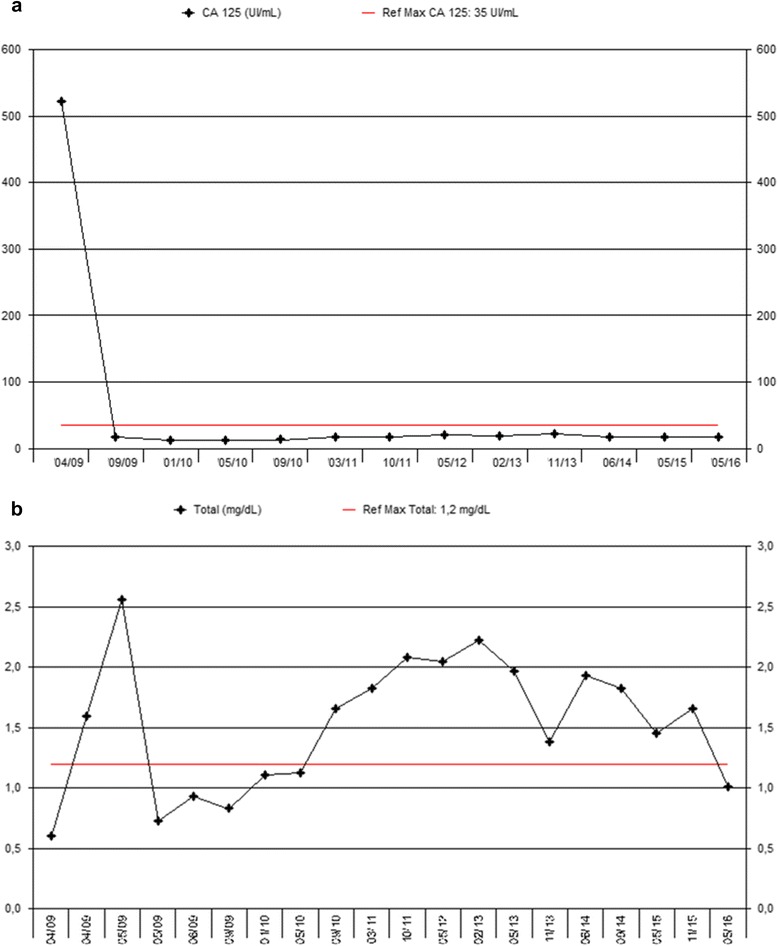
**a** Evolution of Ca-125 levels. **b** Evolution of bilirubin levels

Ten months after the initiation of AIT, she was re-evaluated with a PET/CT scan. A decrease in the size and metabolic activity of the pelvic tumor uptake was observed (SUV max = 5.58), as well as a disappearance of metastatic lymph nodes in the para-aortic region and the uptake of L3 to L4 vertebral bodies (Fig. 6). Her Ca-125 and alkaline phosphatase were 12.6 UI/ml and 141 UI/L, respectively.

Once the excellent objective primary lesion response, the absence of secondary disease, and the significant improvement in PS were established, she was deemed to be a candidate for salvage surgery and an exploratory laparoscopy was performed. During the surgical procedure, the complete removal of the residual mass was considered feasible. The intervention was then converted to a laparotomy and a total hysterectomy with oophorectomy, external iliac lymphadenectomy, a partial cystectomy, excision of small intestine segment, as well as partial debulking of a pelvic residual mass was accomplished. Pathologic examination reported marked bleeding and necrotic changes due to prior treatment, without evidence of tumor disease, and tumor-free lymph nodes (ypT0 ypN0), along with ovarian fibrotic changes secondary to previous AIT, and small bowel atrophy and fibrosis (Fig. 2e).

After the surgery, adjuvant letrozole was restarted with the plan to complete 5 years. Nine months after resection, she presented a major midline abdominal hernia. She required an abdominal eventroplasty. During surgery the right ureteral stent was also removed.

In the following months, she developed a slight but progressive increase in bilirubin levels (from 1.6 to 2.2 mg/dl), which was attributed to the AIT. Therefore, her AIT dose was reduced to 50% and administered every 48 hours, with subsequent normalization of bilirubin levels (Fig. 4b). Letrozole at the reduced dose was maintained until completion of 5 years with good tolerance and an excellent PS, and AIT was discontinued.

After more than 7 years of follow-up, she remains free of disease, with negative Ca-125 tumor marker and without symptoms related to the prior illness or the local and systemic treatments received.

## Discussion

We describe the case of a patient with a metastatic UCS and a dreadful medical condition who not only recovers from the critical initial situation, but also achieves a pathological complete response. Moreover, she maintained long-term disease control without limiting side effects after completing a multimodality therapy with RT, surgery, and 5 years of treatment with AIT.

It must be emphasized that the RT dose administered was 25 Gy. This treatment was given with a palliative intention, and its therapeutic goal was merely hemostatic. To the best of our knowledge, such a low radiation dose has never been demonstrated to have any relevant antitumor activity in uterine sarcoma. Results from evaluation of RT in the adjuvant setting have reported superior local control rates, although no survival advantage has been demonstrated [11]. Moreover, when given postoperatively, the recommended dose for external beam RT is usually 45 Gy. Consequently, it is difficult to assume that a RT dose of 25 Gy may induce the response attained in the massive uterine disease, as well as the lymph nodes, and bone metastases.

After achieving partial remission of uterine disease, our patient underwent salvage surgery. The intervention was limited to the primary uterine tumor. A pathological report did not show any residual tumor and a complete response was concluded from the resected specimen evaluation. Based on these results as well as on the metabolic response observed in the PET/CT scan, no additional RT was administered on the surgical bed, pelvic nodes, and bone metastases.

In metastatic uterine sarcomas, chemotherapy is the most commonly accepted treatment, regardless of its histological type [4, 5, 8]. Other approaches like endocrine therapy can be considered a reasonable management due to the occasional presence of estrogen and progesterone receptors in the neoplastic cells, as well as to the results obtained with AIT as previously described [20–22]. However, it must be taken into consideration that some stablished guidelines for sarcoma treatment like the ESMO Clinical Practice Guidelines indicate that tamoxifen must not be recommended in women with uterine sarcoma, due to its clearly stablished side effects [23]. Likewise, the development of UCS has been reported in series of patients with breast cancer treated with long-term tamoxifen [24]. Other agents including progestins have been successfully used in the treatment of patients with endometrial stromal cell cancer, with response rates ranging from 76 to 88% in different clinical studies on uterine sarcoma using aromatase inhibitors [5]. Patients with metastatic leiomyosarcoma have also been treated with aromatase inhibitors, showing encouraging results [22]. Estrogen receptor expression by tissue microarray analysis in advanced UCS has been found to be enlarged and there was increased disease progression [25]. Despite these facts, aromatase inhibitors have not been demonstrated to improve survival and therefore additional clinical and translational research must be completed to consider these agents active in UCS therapy.

The prognosis of our patient was dismal due to the aggressiveness of the disease and its extension. The administration of systemic chemotherapy was totally contraindicated due to her poor PS and concurrent medical condition, including bleeding, hypovolemic shock, pulmonary thromboembolism, and renal insufficiency. In this context, and given the positivity of estrogen and progesterone receptors in the tumoral cells, the option to initiate oral hormonal therapy with palliative intention seemed reasonable.

## Conclusions

Uterine sarcoma requires a multidisciplinary approach, but the gold standard in advanced disease still has to be defined. Future studies are required to elucidate whether biological and/or targeted therapy has a role in the treatment of these tumors. In the case of metastatic UCS, chemotherapy and palliative symptomatic RT might be helpful in selected cases but, as our case illustrates, aromatase inhibitors should also be considered an active option in these patients.

## Abbreviations

AIT: Aromatase inhibitor therapy
Ca-125: Cancer antigen-125
CT: Computed tomography
ECOG: Eastern Cooperative Oncology Group
PET: Positron emission tomography
PS: Performance status
RT: Radiotherapy
SUV max: Maximum standardized uptake value
UCS: Uterine carcinosarcoma

## References

[1] Major FJ, *et al*. Prognostic factors in early-stage uterine sarcoma. A Gynecologic Oncology Group study. Cancer. 1993;71(4 Suppl):1702–9. 10.1002/cncr.2820710440 8381710

[2] Giuntoli 2nd RL, *et al*. Retrospective review of 208 patients with leiomyosarcoma of the uterus: prognostic indicators, surgical management, and adjuvant therapy. Gynecol Oncol. 2003;89(3):460–9. 10.1016/s0090-8258(03)00137-9 12798712

[3] D’Angelo E, Prat J. Uterine sarcomas: a review. Gynecol Oncol. 2010;116(1):131–9. 10.1016/j.ygyno.2009.09.023 19853898

[4] Pink D, *et al*. Harm or benefit of hormonal treatment in metastatic low-grade endometrial stromal sarcoma: single center experience with 10 cases and review of the literature. Gynecol Oncol. 2006;101(3):464–9. 10.1016/j.ygyno.2005.11.010 16368128

[5] Amant F, *et al*. Clinical management of uterine sarcomas. Lancet Oncol. 2009;10(12):1188–98. 10.1016/S1470-2045(09)70226-8 19959075

[6] Tavassoli F, Devilee P, Organization WH. Tumours of the Breast and Female Genital Organs – Pathology and Genetics. World Health Organization Classification of Tumours. Lyon: IARC Press; 2003.

[7] D’Angelo E, Spagnoli LG, Prat J. Comparative clinicopathologic and immunohistochemical analysis of uterine sarcomas diagnosed using the World Health Organization classification system. Hum Pathol. 2009;40(11):1571–85. 10.1016/j.humpath.2009.03.018 19540555

[8] Reichardt P. The treatment of uterine sarcomas. Ann Oncol. 2012;23 Suppl 10:x151–7. 10.1093/annonc/mds359 22987952

[9] Abeler VM, *et al*. Uterine sarcomas in Norway. A histopathological and prognostic survey of a total population from 1970 to 2000 including 419 patients. Histopathology. 2009;54(3):355–64. 10.1111/j.1365-2559.2009.03231.x 19236512

[10] Dionigi A, *et al*. Endometrial stromal nodules and endometrial stromal tumors with limited infiltration: a clinicopathologic study of 50 cases. Am J Surg Pathol. 2002;26(5):567–81. 10.1097/00000478-200205000-00003 11979087

[11] Wright JD, *et al*. The role of radiation in improving survival for early-stage carcinosarcoma and leiomyosarcoma. Am J Obstet Gynecol. 2008;199(5):536. e1–8. 10.1016/j.ajog.2008.04.019 18511017

[12] Huang GS, *et al*. Serum CA125 predicts extrauterine disease and survival in uterine carcinosarcoma. Gynecol Oncol. 2007;107(3):513–7. 10.1016/j.ygyno.2007.08.060 PMC2696225 17935762

[13] Callister M, *et al*. Malignant mixed Mullerian tumors of the uterus: analysis of patterns of failure, prognostic factors, and treatment outcome. Int J Radiat Oncol Biol Phys. 2004;58(3):786–96. 10.1016/S0360-3016(03)01561-X 14967435

[14] Tanner EJ, *et al*. The role of cytoreductive surgery for newly diagnosed advanced-stage uterine carcinosarcoma. Gynecol Oncol. 2011;123(3):548–52. 10.1016/j.ygyno.2011.08.020 21945551

[15] Gonzalez Bosquet J, *et al*. The impact of multi-modal therapy on survival for uterine carcinosarcomas. Gynecol Oncol. 2010;116(3):419–23. 10.1016/j.ygyno.2009.10.053 19896181

[16] Sutton G, *et al*. A phase III trial of ifosfamide with or without cisplatin in carcinosarcoma of the uterus: A Gynecologic Oncology Group Study. Gynecol Oncol. 2000;79(2):147–53. 10.1006/gyno.2000.6001 11063636

[17] Lacour RA, *et al*. A phase II trial of paclitaxel and carboplatin in women with advanced or recurrent uterine carcinosarcoma. Int J Gynecol Cancer. 2011;21(3):517–22. 10.1097/IGC.0b013e31820da9e2 21436700

[18] Gerszten K, *et al*. The impact of adjuvant radiotherapy on carcinosarcoma of the uterus. Gynecol Oncol. 1998;68(1):8–13. 10.1006/gyno.1997.4901 9454652

[19] Cantrell LA, Blank SV, Duska LR. Uterine carcinosarcoma: A review of the literature. Gynecol Oncol. 2015;137(3):581–8. 10.1016/j.ygyno.2015.03.041 25805398

[20] Spano JP, *et al*. Long-term survival of patients given hormonal therapy for metastatic endometrial stromal sarcoma. Med Oncol. 2003;20(1):87–93. 10.1385/MO:20:1:87 12665689

[21] Reich O, Regauer S. Hormonal therapy of endometrial stromal sarcoma. Curr Opin Oncol. 2007;19(4):347–52. 10.1097/CCO.0b013e3281a7ef3a 17545798

[22] O’Cearbhaill R, *et al*. Treatment of advanced uterine leiomyosarcoma with aromatase inhibitors. Gynecol Oncol. 2010;116(3):424–9. 10.1016/j.ygyno.2009.10.064 PMC4852374 19932916

[23] ESMO/European Sarcoma Network Working Group. Soft tissue and visceral sarcomas: ESMO Clinical Practice Guidelines for diagnosis, treatment and follow-up. Ann Oncol. 2014;25 Suppl 3:iii102–12. 10.1093/annonc/mdu254 25210080

[24] McCluggage WG, *et al*. Uterine carcinosarcoma in association with tamoxifen therapy. Br J Obstet Gynaecol. 1997;104(6):748–50. 10.1111/j.1471-0528.1997.tb11992.x 9197885

[25] Huang GS, *et al*. Tissue microarray analysis of hormonal signaling pathways in uterine carcinosarcoma. Am J Obstet Gynecol. 2009;200(4):457. e1–5. 10.1016/j.ajog.2008.12.012 PMC2676778 19200930

